# A quantitative method to decompose SWE differences between regional climate models and reanalysis datasets

**DOI:** 10.1038/s41598-019-52880-5

**Published:** 2019-11-11

**Authors:** Yun Xu, Andrew Jones, Alan Rhoades

**Affiliations:** Lawrence Berkeley National Laboratory, Earth and Environment Sciences Area, Berkeley, CA 94720 USA

**Keywords:** Cryospheric science, Cryospheric science, Hydrology, Hydrology

## Abstract

The simulation of snow water equivalent (SWE) remains difficult for regional climate models. Accurate SWE simulation depends on complex interacting climate processes such as the intensity and distribution of precipitation, rain-snow partitioning, and radiative fluxes. To identify the driving forces behind SWE difference between model and reanalysis datasets, and guide model improvement, we design a framework to quantitatively decompose the SWE difference contributed from precipitation distribution and magnitude, ablation, temperature and topography biases in regional climate models. We apply this framework within the California Sierra Nevada to four regional climate models from the North American Coordinated Regional Downscaling Experiment (NA-CORDEX) run at three spatial resolutions. Models generally predict less SWE compared to Landsat-Era Sierra Nevada Snow Reanalysis (SNSR) dataset. Unresolved topography associated with model resolution contribute to dry and warm biases in models. Refining resolution from 0.44° to 0.11° improves SWE simulation by 35%. To varying degrees across models, additional difference arises from spatial and elevational distribution of precipitation, cold biases revealed by topographic correction, uncertainties in the rain-snow partitioning threshold, and high ablation biases. This work reveals both positive and negative contributions to snow bias in climate models and provides guidance for future model development to enhance SWE simulation.

## Introduction

Snowpack is a key component of the Earth’s climate. It affects the earth’s energy balance through high albedo and low heat conductivity, and in the mountains acts as a natural reservoir to store water in winter and slowly release meltwater in summer. More than one-sixth of the world’s population relies on seasonal snow for water^1^. Rising air temperatures reduce snowfall and enhance snow melt in spring and summer, resulting in a decline in the volume and duration of mountain snowpack, earlier peak snow mass timing, and therefore a deficit of summer water supply^1–3^. Observations have revealed a declining mountain snowpack in the western US over the last half-century due to climate warming^4–6^. The depletion of snowpack increases fire potential^7^, alters natural ecosystem^8^, and affects the Earth’s energy budget^9^.

Numerical models offer promise to predict future changes in snowpack and to inform the interactions between snowpack and other climate components. The accurate simulation of seasonal snow dynamics in mountainous regions requires that models correctly represent a number of interacting processes including storm track location, landfall and timing, orographic uplift of storms and accurate dispersal of precipitation over the mountain. Moreover, radiation, temperature and moisture fluxes at the snow surface must be accurate to ensure that the residence time of snowpack is correct. Therefore, snow simulations can serve as a crucial litmus test for climate model performance since their accuracy requires so many processes to be represented with fidelity. However, the evaluation of snowpack in regional climate models has not received as much attention as other variables such as temperature, precipitation, and radiation.

Climate model evaluations related to snow have often focused on total precipitation with no rain-snow distinction, or on snow cover for which accurate measurements exist for evaluation^10,11^. The snow water equivalent (SWE), the measure of water volume contained within the snowpack, has received less attention in part because of the lack of reliable observational datasets for comparison with models. *In-situ* observations suffer from large uncertainties from interpolation when comparing with gridded model outputs, and re-analysis or remote sensing datasets highly disagree from one another^12^. A few studies that managed to evaluate SWE simulations have discovered significant SWE bias in climate models^13–15^. The poor performance of SWE simulations highlights the need to investigate the cause of SWE bias to assist model development.

The complex drivers of snow dynamics mean that biases could cancel one another out and mask the true drivers of model uncertainty. Quantitative methods for decomposing the drivers of snowpack simulation performance are needed to disentangle interacting biases and identify critical areas for model improvement. However current studies on SWE simulation diagnosis are mostly limited to empirical qualitative estimates^14,16^ or sensitivity experiments confined within standalone land surface models^17^. Traditional quantitative estimation of uncertainty contribution is to run sensitivity experiments repeatedly, which is not computationally affordable for complex regional climate models especially when there are multiple potential causal variables to test.

**Figure 1:**
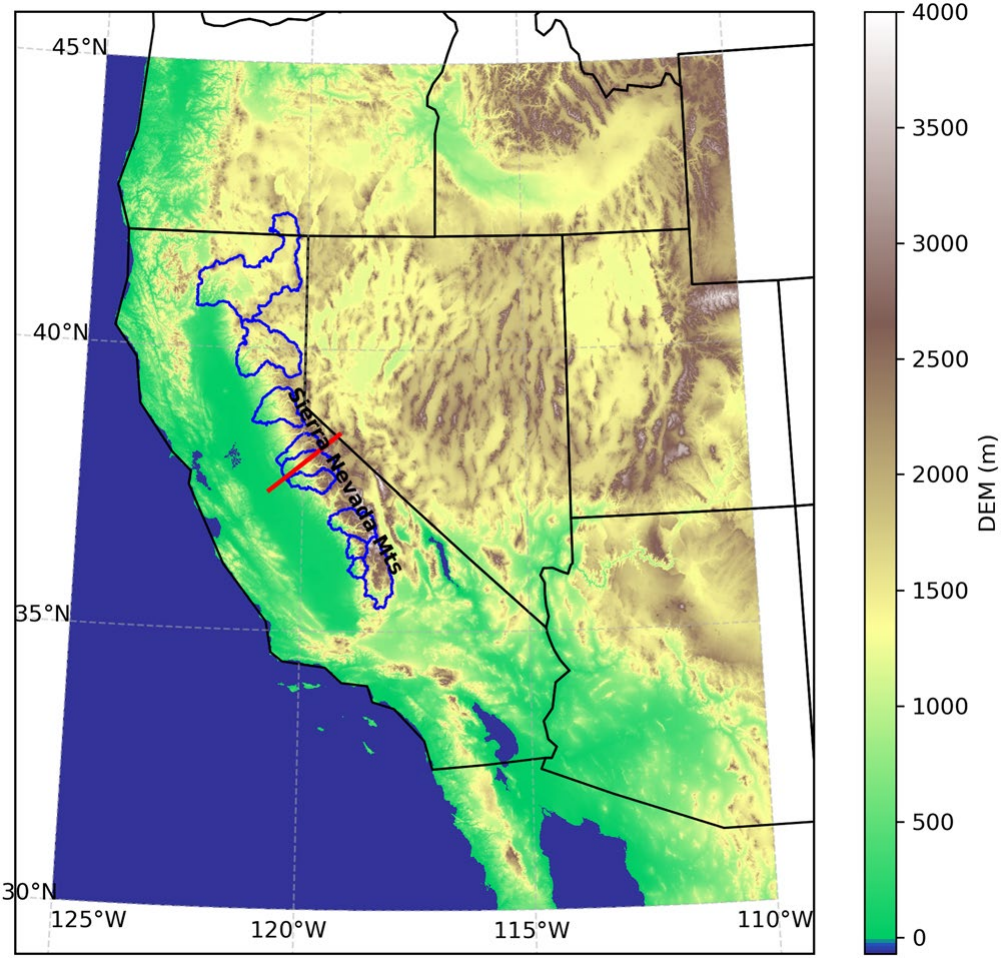
Map of the study area in the western United States. The colored map shows the topography elevation from a digital elevation model (DEM) from the Global 30 Arc-Second Elevation (GTOPO30)^57^. The 10 California headwater regions that are evaluated in this study are outlined in blue. The red line that transects the Sierra Nevada mountain range is used to compare the reference and model results in Supplementary Fig. S1.

Here we develop a framework to quantitatively diagnose which factors and processes contribute to the different estimates of SWE between reanalysis and regional climate models. We decompose the difference associated with temperature, precipitation, and ablation dynamics through the cascade of atmosphere-land processes. We apply this framework to decompose SWE difference from reanalysis in North American Coordinated Regional Downscaling Experiment (NA-CORDEX) simulations^18^, which provide a rich dataset to examine given that it contains multiple regional climate models (RCMs) at multiple resolutions. We focus on the California Sierra Nevada where the Landsat-Era Sierra Nevada Snow Reanalysis (SNSR) dataset^19^ provides highly accurate and high resolution SWE data for reference, with a particular focus on 10 watershed regions (Fig. 1) that directly feed into 10 major reservoirs of the State^15,20^. Since the observation-based datasets used as reference quantities are themselves imperfect descriptions of reality, we further quantify the uncertainty in our quantitative decomposition framework that results from published uncertainty estimates for the reference datasets.

## Results

**Figure 2:**
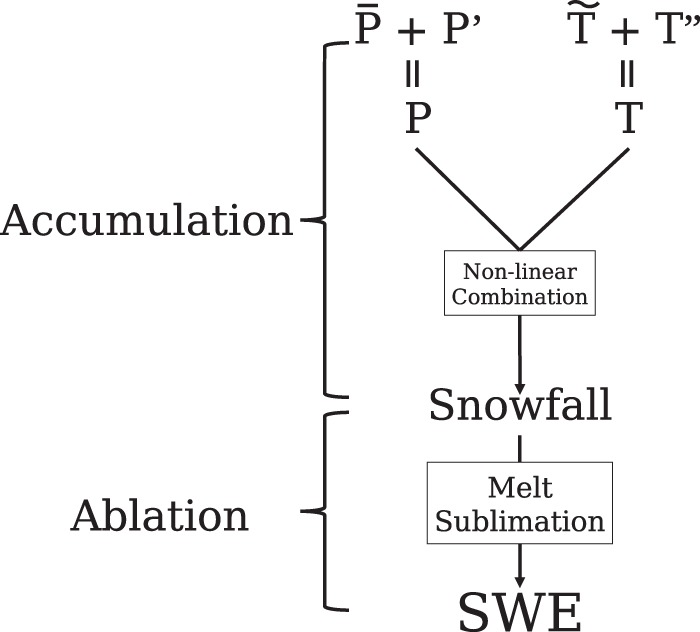
Climate variables that impact snow water equivalent (SWE). SWE is determined by the amount of snowfall, or accumulation, and the amount of snow melt and sublimation, or ablation. Snowfall results from non-linear combination of temperature (*T*) and precipitation (*P*). These non-linear interactions can be further partitioned into mean *P* ($\bar{P}$) plus the distribution (${P}^{{\rm{^{\prime} }}}$) and the topographic influence on *T* ($\mathop{T}\limits^{ \sim }$) and the residual topography corrected (${T}^{{\rm{^{\prime} }}{\rm{^{\prime} }}}$).

**Figure 3:**
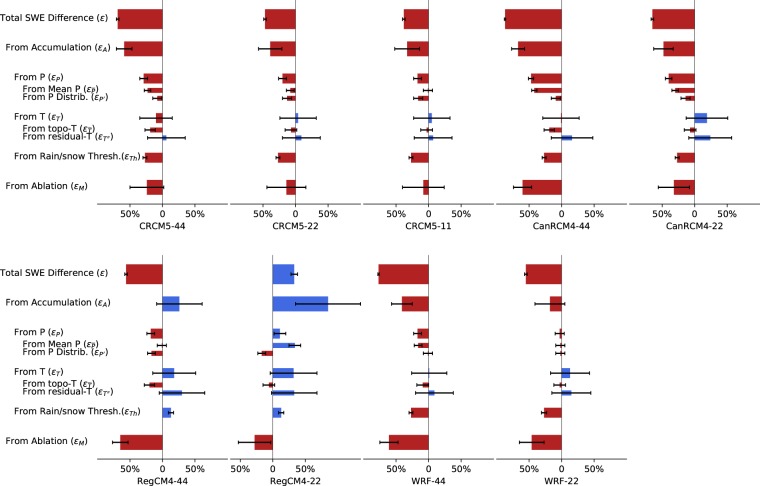
Quantitative decomposition of the model-reference difference in climatological March 1 SWE across reanalysis driven RCM simulations from NA-CORDEX. Colour bars indicate the percentage bias in SWE contributed from each source, and the ticks indicate 95% confidence intervals associated with each of the estimated percentage biases. The vertical thickness of each bar reflects its order within the hierarchy of quantitative decomposition, with successive layers of decomposition corresponding to thinner bars. Simulations are named in the format of RCM-resolution. The four RCMs include CRCM5, CanRCM4, RegCM4 and WRF. Resolution labeled as 44, 22 and 11 means horizontal grid spacing of 0.44°, 0.22° and 0.11°.

As shown in Fig. 2, the annual cycle of SWE is shaped by snow accumulation (*A*) and snow ablation (*M*). Snow ablation is shaped by both snow melt and sublimation. Snow accumulation is the amount of snowfall (*S*) that depends on precipitation (*P*) and temperature (*T*): *P* that occurs below a rain-snow threshold temperature (*Th*) is snow. The interplay between precipitation and temperature are influenced by many factors such as the model’s parameterizations, topography, and model resolution. To investigate these factors, *P* is further decomposed into its mean ($\bar{P}$) and the distribution ($P^{\prime}$), and *T* is further partitioned into topography-related ($\tilde{T}$) and the topography-corrected ($T^{\prime\prime}$) components. Topography-related $\tilde{T}$ is computed by taking the reference *T* and modifying it to include model topography misrepresentation, and topography-corrected $T^{\prime\prime}$ is calculated by correcting modeled *T* using the reference topography. These climate variables are compared between reference datasets and nine reanalysis driven NA-CORDEX simulations including four RCMs running at three spatial resolutions of 0.44°, 0.22° and 0.11°. The four RCMs are: Canadian Centre for Climate Modelling and Analysis Regional Climate Model^21^ (CanRCM4), the fifth-generation of the Canadian Regional Climate Model version 5^22–24^ (CRCM5), Regional Climate Model version 4^25^ (RegCM4), and Weather Research and Forecasting model (WRF)^26^. Metrics are designed to quantitatively decompose the total model-reanalysis difference ($\varepsilon$) in representing peak SWE to the relative contribution from each of the causal variables. The SWE difference contributed from a causal variable, denoted as ${{\rm{\varepsilon }}}_{{\rm{X}}}$ with X standing for any of the causal variables mentioned above, is calculated as the percentage change in peak SWE when X is changed from the reference to model values. Uncertainties in reference T, P, SWE and rain-snow partitioning influence how certain we are about ${{\rm{\varepsilon }}}_{{\rm{X}}}$, as such we add 95% confidence intervals to each value in Fig. 3 to evaluate if the contribution from each causal variable is significantly different from 0.

**Figure 4:**
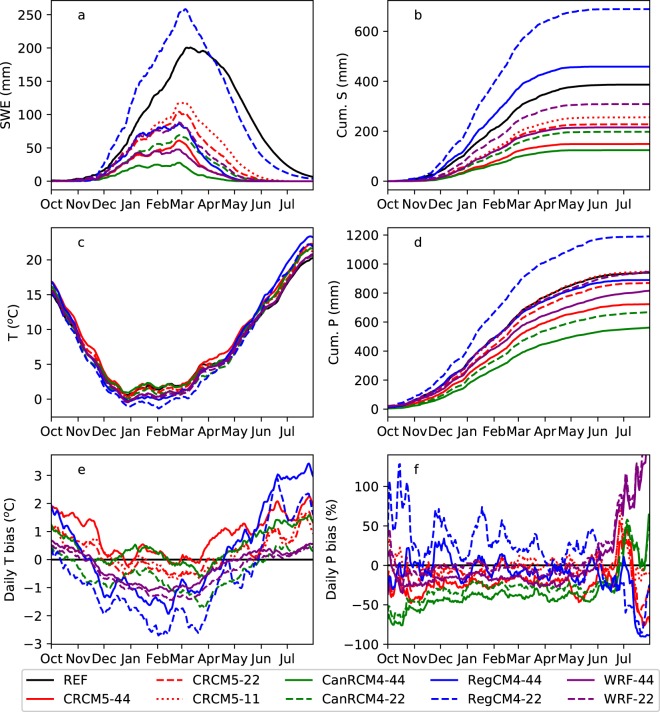
Climatological mean of (**a**) SWE, (**b**) cumulative snowfall (Cum. S), (**c**) daily mean temperature (T), (**d**) cumulative precipitation (Cum. P), (**e**) bias of daily mean T (T bias) and (**f**) bias of daily P (P bias) averaged over 10 headwater regions for both NA-CORDEX simulations and reference datasets (REF). T bias is the absolute difference in °C between model and reference, while P bias is the percentage difference. Cumulative P (S) are defined as the cumulative sum of daily P (S) from October 1^st^.

Figure 4 compared the climatological mean of *P*, *T*, *S* and *SWE* over 20 water years spanning October 1989 to September 2009, averaged over the 10 watershed regions. Most models, except RegCM4–22, underestimate the peak SWE value, $\widehat{SWE}$. Because the climatological peak SWE date occurs around March 1^st^ in both the reference and simulation datasets, we use March 1^st^ as the nominal peak SWE date for all datasets, and therefore $\widehat{SWE}$ is the SWE on March 1^st^ hereafter. Reference $\widehat{SWE}$ is 190 ± 6 mm (Supplementary Table S1). RegCM4–22 overestimates $\widehat{SWE}$ by + 33%, and all other models underestimate $\widehat{SWE}$ by −87% to −38%, with 1–5% uncertainty due to the reference SWE. The percentage bias in $\widehat{SWE}$ defines the model total bias in $\widehat{SWE}$ ($\varepsilon$), and the values are shown in Fig. 3. Running at the same spatial resolution, RegCM4 predicts the highest values of SWE, CanRCM4 predicts the lowest, and CRCM5 and WRF show similar values in between. All four RCMs predict higher SWE values with finer spatial resolution. Compared to the reference dataset a systemically shorter snow season duration and abrupt snow melt is found across all RCMs which is corroborated and discussed in more detail in previous studies^15,20^.

The cumulative *S* from October 1^st^ to March 1^st^ ($\hat{S}$) is 294 mm in the reference dataset (Fig. 4b). The simulation of *S* contains large bias, and the percentage bias in $\hat{S}$ equals the accumulation induced $\widehat{SWE}$ difference (${{\rm{\varepsilon }}}_{{\rm{A}}}$) as derived in the Methods section. Even though large uncertainties exist, we find overestimation of $\hat{S}$ in RegCM4 and underestimation in other models (Fig. 3). Underestimation is most significant in CanRCM4, followed by CRCM5 and then WRF. In agreement with SWE, predicted *S* increases with finer model resolution.

The ablation rate (*M*), defined as the percentage of $\hat{S}$ lost before March 1^st^, is 35% in the reference data with ±22% uncertainty resulted from the uncertainties in *SWE* and in *S* (Supplementary Table S1). CanRCM4, RegCM4 and WRF significantly overestimate snow ablation, which results in underestimation of *S* (Fig. 3, row ${\varepsilon }_{M}$). CRCM5 performs better than other models with insignificant overestimation of ablation. In general, simulated ablation improves with finer model resolution.

All of the models are able to represent the seasonal variation of *T* (Fig. 4c), but tend to underestimate winter *T* by −2.7 to −0.2 °C and overestimate summer *T* by + 0.5 to + 3.4 °C (Fig. 4e). Models run at finer resolution tend to generate lower *T*. Across all models, the RegCM4 model produces the highest bias in both winter and summer *T* compared to other models. Uncertainties in reference *T*^27^ lead to large uncertainties when evaluating the *T* induced $\widehat{SWE}$ difference (${\varepsilon }_{T}$). Nevertheless, we find the cold bias in winter *T* in RegCM4 led to an insignificant overestimation of $\widehat{SWE}$ by 18% to 32%, whereas in other models ${\varepsilon }_{T}$ of −10% to 19% is found (Fig. 3). The model bias in topography leads to a warm bias (Supplementary Fig. S1) and therefore the underestimation of $\widehat{SWE}$ by −20% to −3% in all resolutions (Fig. 3, row ${\varepsilon }_{\tilde{T}}$). The ${\varepsilon }_{\tilde{T}}$ decreases with increased refinement of model resolution as topography is better represented. All models run at the same resolution have similar values of ${\varepsilon }_{\tilde{T}}$, indicating the accuracy of topography is highly related to model resolution. After correction for topography, $T^{\prime\prime}$ contributes to an overestimation of $\widehat{SWE}$ by 6% to 33% (Fig. 3, row ${\varepsilon }_{T^{\prime\prime} }$). The value of ${\varepsilon }_{T^{\prime\prime} }$ is insensitive to the change of resolution, but varies between models.

The simulation of *P* is similar in character to *S* (Fig. 4d). The reference dataset suggests that cumulative *P* from October 1^st^ to March 1^st^ is 636 mm. RegCM4–22 overestimates this value by 34%, and all other simulations underestimate the value by up to −42%. Simulated *P* increases with resolution. High resolution simulations by CRCM5 and WRF converge cumulative *P* to the reference. The cumulative *P* is the spatial average of the surveyed regions, and the difference in cumulative *P* equals the $\bar{P}$ induced $\widehat{SWE}$ difference as shown in Fig. 3, row ${\varepsilon }_{\bar{P}}$. To calculate the total $\widehat{SWE}$ difference introduced by *P* (${\varepsilon }_{P}$), we find an overestimation of $\widehat{SWE}$ in RegCM4–22, and significant underestimation in other models except WRF-22 and RegCM4–44 (Fig. 3, row ${\varepsilon }_{p}$). After correction for mean *P*, the residual $P^{\prime}$ still contributes to significant underestimation of $\widehat{SWE}$, except in WRF simulations (Fig. 3, row ${\varepsilon }_{p^{\prime} }$).

**Figure 5:**
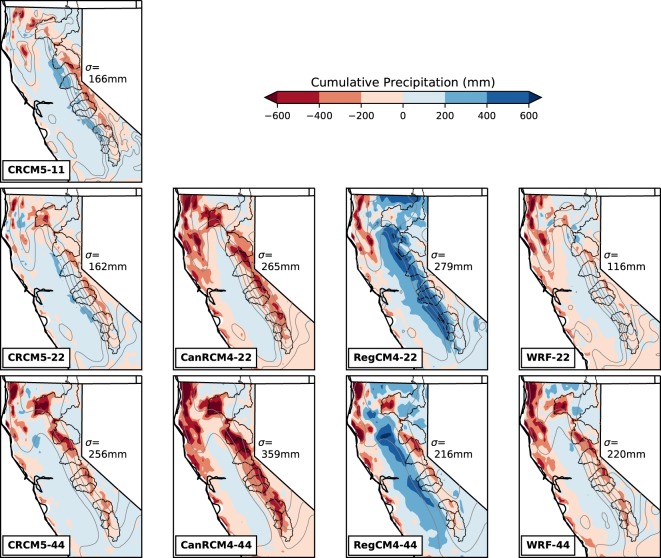
The model bias in multi-year mean winter cumulative precipitation (mm) from October 1^st^ to March 1^st^ compared with reference. Root-mean-square of the model-reference difference in each simulation is labeled σ in each subplot. Topography is shown by gray contours with 500 m interval. The boundary of the 10 headwater regions are shown by black outlines.

The spatial distribution of the multi-year mean *P* illustrates how simulated $P^{\prime}$ leads to $\widehat{SWE}$ underestimation (Fig. 5). The $P^{\prime}$ represents the spatial and temporal distribution of *P*, but because the temporal pattern of $P^{\prime}$ shows no obvious trend in winter months (Fig. 4 f), $P^{\prime}$ mainly indicates the spatial distribution of *P*. Most of the NA-CORDEX models either underestimate *P* at high-elevation or overestimate it at mid-elevation or both. The accuracy of *P* is improved at high resolution (except RegCM4), but a common tendency for models to simulate a dry bias at the mountain crest and wet bias at mid-elevation remains even in the highest resolution simulation (e.g., CRCM5–11). Moving some *P* from the relatively colder mountain crest to relatively warm mid-elevation did not favor the formation of S which results in less SWE accumulation.

RegCM4 assumes precipitation occurs below a rain-snow threshold temperature (*Th*) of 2.2 °C is all snow, and other models assume *Th* is 0 °C. To better account for observed rain-snow partitioning in the Sierra Nevada, the reference data is assumed to have a linear change of snow percentage from 0–3 °C^28,29^. The difference in *Th* introduces an additional −27% underestimation in $\widehat{SWE}$ in CRCM5, CanRCM4 and WRF, and 13% overestimation in $\widehat{SWE}$ in RegCM4 (Fig. 3, row ${\varepsilon }_{Th}$).

## Discussion

We have shown that all the surveyed numerical models show large SWE difference from reference dataset in the California Sierra Nevada. While all the models overestimate snow ablation, different models perform differently in simulating snow accumulation processes. RegCM4 displays a wetter and colder climate compared to other models, and allows snow formation in a warmer atmosphere, and therefore predicts much more snowfall than other models. CanRCM4 shows the biggest dry bias compared to other models. CRCM5 performs better than CanRCM4 in all aspects including precipitation, temperature and ablation. Mean precipitation in CRCM5 converges to the reference at 0.11° resolution. The lowest ablation difference from reference is found in CRCM5, potentially due to the improved treatment of snow processes in the land surface model (LSM) such as improved treatment of snow albedo, liquid water retention and vertical temperature gradient^30,31^. WRF outperforms other models in snow accumulation processes, especially the mean and spatial distribution of precipitation, but not on snow ablation, likely due to known biases in the Noah LSM^14,32–34^. Though there have been many studies to evaluate temperature and precipitation simulations over North America^35,36^, our work reveals more details in California’s mountain regions with particular interest in the climate processes related to snowpack formation.

With increasing spatial resolution, the models show a wetter and colder state. Low spatial resolution is unable to resolve complex topography in the California Sierra Nevada, and the mountain elevation could be up to 1 km too low, corresponding to a 5 °C warm bias at particular locations as shown in Supplementary Fig. S1. The lower mountains also limit the condensation of water vapor and therefore the amount of precipitation. Conversely, increasing resolution resolves more small-scale features and further increases precipitation^37,38^. As a result, increasing model resolution from 0.44° to 0.11° improves the simulated SWE by 35% through the improvement of mean precipitation and topography-related temperature.

However, our analysis reveals that significant biases remain even at high resolution. Many studies have found that precipitation does not converge with high resolution^39–41^. Our analysis also shows that increasing spatial resolution from 0.44° to 0.11° brings the temperature induced model-reanalysis SWE difference from negative to positive rather than converging to the reference. By decomposing the temperature, we find a topography-related warm bias that does converge toward zero with higher resolution (Fig. 3, row “From Topo-T”). This in turn reveals an underlying cold bias that is unrelated to resolution (Fig. 3, row “From residual-T”). The temperature induced SWE bias contains large uncertainties due to the uncertainty in the reference *T* (see Supplementary Note for details). However, if the reference *T* is cold biased as discovered in the leeward side of Sierra Nevada^27^, this further demonstrates the cold bias in $T^{\prime\prime}$ and the resultant overestimation of SWE. This analysis demonstrates the value of our decomposition framework for assessing how positive and negative biases can mask one another and lead to false positives in model skill.

Cold bias in $T^{\prime\prime}$ is consistent in all the models. This seems to be related to the model’s underestimation of winter temperature. Although the colder winters simulated by the models increase the probability that *S* can occur, it appears that the large ablation biases led to the underestimation of $\widehat{SWE}$ and was further enhanced by a warmer spring and summer leading to abrupt snow melt and a shortened snow season^15^. The seasonal temperature biases in the regional climate models can be introduced by the input boundary conditions from the reanalysis dataset. However, the same seasonality of temperature bias is also found in the RCMs forced by different boundary conditions from global climate models (Supplementary Fig. S2) and in many other LSMs with various atmospheric forcings^13^. Other possible reasons for the seasonality of temperature bias could be the regional climate model’s inherent structural and/or parameter bias or more specifically biases associated with the snow albedo feedback. For example, Noah LSM assumes 100% snow cover when SWE exceeds a certain threshold, which would result in overestimated snow cover and hence cold bias in winter^42^; using average albedo in each grid cell leads to lower snow surface albedo when snow cover is less than 100%, and hence extra absorption of insolation and warm bias in spring^13^.

We have shown that even after correcting for the mean precipitation, there is still an underestimation of SWE in CRCM5, CanRCM4 and RegCM4 models, resulting from the biased spatial distribution of precipitation. In particular, precipitation at cold mountain tops is underestimated in favor of relatively warm mid-elevation. One possible mechanism for the biased precipitation distribution is the microphysics scheme^41^. The models may not represent horizontal transport of rain droplets and/or snowflakes with wind appropriately, which can produce too much precipitation at particular elevation bands. Another possible explanation could be the hydrostatic assumption in the models, which limits development of convection over mountain regions^43^. Supporting this explanation is the fact that the sole non-hydrostatic model we considered (WRF) has a P-distribution induced model-reanalysis SWE difference (${\varepsilon }_{p\text{'}}$) that is one order of magnitude smaller than other models. However, there are many other differences in the model configuration between WRF and the other models in NA-CORDEX project, so we cannot eliminate other possible causes for the biased precipitation distribution.

Jennings *et al*.^44^ suggest a highly variable rain-snow partitioning over the Northern Hemisphere dependent on humidity, topography and climate type. Rain-snow thresholds in most LSMs are solely temperature based, yet humidity is an important factor in maintaining snow from the cloud to the surface. This is explained by the hydrometeor energy balance theory where low ambient relative humidity promotes evaporative cooling via exchanges in latent heat which enables snowflakes to remain frozen in above freezing environments. Our reference rain-snow partitioning schema assumes a linear change in snowfall percentage between 0°C and 3°C, based on observations made in the Sierra Nevada^28,29^, and therefore might not be applicable to other regions. However, the simplified rain-snow threshold across models led to a significant bias in SWE for the Sierra Nevada and should be noted for model simulations in the future.

Our results reveal large bias in modeled snow ablation, which is consistent with an earlier study^15^ that also revealed significant overestimation of snow ablation in spring and summer. Even though the cold bias in winter tends to underestimate snow ablation, the overestimation of snow ablation could result from many factors^45^, such as overestimated downwelling longwave and shortwave irradiance^46,47^, overestimated downward sensible and latent heat flux resulting in high snow sublimation^48^, biased snow-albedo decay, liquid water refreeze schema, the selection of area snow depletion thresholds^30,33^, misrepresentation of wind redistribution and turbulent mixing above the snowpack^49^ and/or the uncertainty in rain-snow partitioning^44^. To investigate the potential causal mechanisms of ablation biases in the context of the present study would require an examination of the simulated surface energy balance across models but these variables are unavailable from NA-CORDEX.

## Conclusions

In this paper, we present a new method to quantitatively decompose simulated snowpack biases into contributing sources from upstream processes and phenomena. We apply this method to an ensemble of NA-CORDEX regional climate model simulations in the California Sierra Nevada and reveal that many of these models largely underestimate California SWE due to a combination of model-reference differences in snow accumulation and ablation, uncertainties in precipitation phase, and unresolved topography. In some cases, positive and negative contributions to SWE biases mask one another. Our method reveals the contribution from each individual source to model-reanalysis SWE difference, and uncovers compensating contributions that would otherwise not be apparent. In this way, the analysis identifies major sources of model-reanalysis difference that need to be improved in order to more accurately represent SWE dynamics. We note that our decomposition of SWE difference between climate models and reference datasets is only as good as our reference datasets used. Therefore, this decomposition framework could be periodically used in future studies as more accurate observational and/or reanalysis datasets are made available.

## Methods

### Decomposing SWE difference between model and reference datasets

In this paper, we design a framework to quantitatively decompose the model total bias in representing peak SWE to the contribution from each of the causal variables as shown in Fig. 2. Total bias in the modeled peak SWE value ($\varepsilon$) is defined as the percentage difference compared with reference:

1 $$\varepsilon =\frac{{\widehat{SWE}}_{cdx}-{\widehat{SWE}}_{ref}}{{\widehat{SWE}}_{ref}}=\frac{{\widehat{SWE}}_{cdx}}{{\widehat{SWE}}_{ref}}-1$$

where $\widehat{SWE}$ stands for the peak SWE value on March 1^st^, the nominal peak SWE date. Subscript “cdx” and “ref” indicate variables from NA-CORDEX simulations and reference datasets respectively.

The contribution from a certain climate variable X to the bias in SWE (${{\rm{\varepsilon }}}_{{\rm{X}}}$) is calculated as percentage change from reference $\widehat{{\rm{SWE}}}$ if variable X_ref_ is substituted by X_cdx_ and all other variables remain the same:

2 $${\varepsilon }_{X}=\frac{{\widehat{SWE}}_{{X}_{cdx}}-{\widehat{SWE}}_{ref}}{{\widehat{SWE}}_{ref}}=\frac{{\widehat{SWE}}_{{X}_{cdx}}}{{\widehat{SWE}}_{ref}}-1$$

where ${\widehat{SWE}}_{{X}_{cdx}}$ is the $\widehat{SWE}$ calculated using variable X from the NA-CORDEX simulation and all other variables from the reference datasets. Below we give specific examples.

We first decompose model-reanalysis difference in $\widehat{{\rm{SWE}}}$ (${\rm{\varepsilon }}$) into accumulation induced ${{\rm{\varepsilon }}}_{{\rm{A}}}$ and ablation induced ${{\rm{\varepsilon }}}_{{\rm{M}}}$ difference. Because snow ablation data was unavailable from NA-CORDEX simulations, we cannot directly compare snow ablation between the reference and NA-CORDEX. Instead, we define the ablation rate *M* as the percentage of *S* that is lost before peak SWE date (March 1st):

3 $$M=1-\widehat{SWE}/\hat{S}$$

where $\hat{S}$ is the cumulative S from October 1^st^ to March 1^st^. The above equation can be rewritten as a function of $\widehat{SWE}$:

4 $$\widehat{SWE}=\hat{S}(1-M)$$

$\widehat{{\rm{SWE}}}$ difference contributed from snow accumulation equals percentage difference in cumulative S:

5 $${\varepsilon }_{A}=\frac{{\widehat{SWE}}_{{S}_{cdx}}}{{\widehat{SWE}}_{ref}}-1=\frac{{\hat{S}}_{cdx}(1-{M}_{ref})}{{\hat{S}}_{ref}(1-{M}_{ref})}-1=\frac{{\hat{S}}_{cdx}}{{\hat{S}}_{ref}}-1$$

and $\widehat{SWE}$ difference contributed from snow ablation equals

6 $${\varepsilon }_{M}=\frac{{\widehat{SWE}}_{{M}_{cdx}}-{\widehat{SWE}}_{ref}}{{\widehat{SWE}}_{ref}}=\frac{{S}_{ref}(1-{M}_{cdx})-{S}_{ref}(1-{M}_{ref})}{{S}_{ref}(1-{M}_{ref})}=\frac{{M}_{ref}-{M}_{cdx}}{1-{M}_{ref}}$$

By substituting Eqs. (4–6) into Eq. (1), we can rewrite $\varepsilon$ as a function of ${\varepsilon }_{A}$ and ${\varepsilon }_{M}$:

7 $$\varepsilon =\frac{{\widehat{SWE}}_{cdx}}{{\widehat{SWE}}_{ref}}-1=\frac{{\hat{S}}_{cdx}}{{\hat{S}}_{ref}}\cdot \frac{1-{M}_{cdx}}{1-{M}_{ref}}-1=({\varepsilon }_{A}+1)({\varepsilon }_{M}+1)-1$$

We then decompose ${\varepsilon }_{A}$ by *P*, *T*, and *Th*. The contribution of *P* to $\widehat{SWE}$ difference (${\varepsilon }_{P}$) is

8 $${\varepsilon }_{P}=\frac{{\widehat{SWE}}_{{P}_{cdx}}}{{\widehat{SWE}}_{ref}}-1=\frac{{\hat{S}}_{{P}_{cdx}}}{{\hat{S}}_{ref}}-1$$

where ${\hat{S}}_{{P}_{cdx}}$ is the $\hat{S}$ calculated using *P* from NA-CORDEX, and all other variables (e.g., *T*) from the reference. Similarly, *T* contribution to $\widehat{SWE}$ difference ${\varepsilon }_{T}$ is

9 $${\varepsilon }_{T}=\frac{{\widehat{SWE}}_{{T}_{cdx}}}{{\widehat{SWE}}_{ref}}-1=\frac{{\hat{S}}_{{T}_{cdx}}}{{\hat{S}}_{ref}}-1$$

Th contribution to $\widehat{SWE}$ difference ${\varepsilon }_{Th}$ is

10 $${\varepsilon }_{Th}=\frac{{\widehat{SWE}}_{T{h}_{cdx}}}{{\widehat{SWE}}_{ref}}-1=\frac{{\hat{S}}_{T{h}_{cdx}}}{{\hat{S}}_{ref}}-1$$

We further derive the $\widehat{SWE}$ difference from $\bar{P}$, $P^{\prime}$, $\tilde{T}$ and $T^{\prime\prime}$ in a similar way using the equation:

11 $${\varepsilon }_{X}=\frac{{\hat{S}}_{{X}_{cdx}}}{{\hat{S}}_{ref}}-1$$

where X could be $\bar{P}$, $P\text{'}$, $\tilde{T}$ and $T^{\prime\prime}$. While $\bar{P}$ is the mean *P*, $P\text{'}$ is the distribution, or *P* corrected for the mean value. Therefore ${\hat{S}}_{{\bar{P}}_{cdx}}$ is calculated using *P*_*ref*_ scaled by ${\bar{P}}_{cdx}/{\bar{P}}_{ref}$, by which we keep the $P\text{'}$ as in reference, but $\bar{P}$ as in NA-CORDEX. ${\hat{S}}_{P{\text{'}}_{cdx}}$ is calculated using *P*_*cdf*_ scaled by ${\bar{P}}_{ref}/{\bar{P}}_{cdx}$ to keep $P\text{'}$ as in NA-CORDEX and $\bar{P}$ as in reference. To derive the $\widehat{SWE}$ difference contributed from topography-related $\tilde{T}$ and topography-corrected $T^{\prime\prime}$, we calculate ${\hat{S}}_{{\tilde{T}}_{cdx}}$ using *T*_*ref*_ but modified to the NA-CORDEX topography and lapse rate, so that the only difference between ${\hat{S}}_{{\tilde{T}}_{cdx}}$ and ${\hat{S}}_{ref}$ is the temperature difference resultant from the misrepresentation of topography in the model. ${\hat{S}}_{\mbox{''}}\,$is calculated using *T*_*cdx*_ but corrected to the reference topography using the same lapse rate. The lapse rate in the windward side of the Sierra Nevada has been shown to vary between 3.5 and 5.0 °C/km^50^. We use the mean value 4.3 °C/km in our calculation, but consider corresponding uncertainties.

### Data

We examine nine NA-CORDEX simulations driven by atmospheric reanalysis data from the European Centre for Medium‐range Weather Forecasts (ECMWF) known as ERA-interim. These simulations were carried out by four RCMs: CanRCM4, CRCM5, RegCM4 and WRF. The models were run over a North American domain at three different spatial resolutions of 0.44°, 0.22° and 0.11°. CanRCM4 and CRCM5 assume hydrostatic equilibrium and use the Canadian land surface scheme version 2.7 and 3.5 (CLASS 2.7 and CLASS 3.5)^51,52^ respectively as the Land Surface Model (LSM). RegCM4 also assumes hydrostatic equilibrium and uses the Biosphere-Atmosphere Transfer Scheme (BATS)^53^ as LSM. WRF implements non-hydrostatic dynamics and uses Noah LSM^54^. *S* in these models is determined by the rain-snow threshold temperature (*Th*), which is 2.2 °C in RegCM4 and 0 °C in the other models. Further details of the RCM characteristics can be found at NA-CORDEX website^18^.

We download daily variables of *SWE*, *T*, *P* and topography from NA-CORDEX simulations, and calculate *S* from *T*, *P* and *Th*. These variables should be compared with ground truth in order to derive model bias contribution from various sources using our decomposition method. However, there is no ground truth available for the comparison. *In-situ* observations represent the status of certain points, while model variables represent the mean status of each grid cell. Remote sensing data suffers from satellite retrieval uncertainty, re-analysis data contains model bias, and gridded data based on *in-situ* data has uncertainty from interpolation. In this paper, we try to select reference datasets to best represent the ground truth, but also acknowledge the associated uncertainties.

The Landsat-Era Sierra Nevada Snow Reanalysis (SNSR) dataset^19^ is chosen as the reference SWE dataset. The SNSR dataset covers the California Sierra Nevada from year 1985 to present day at daily temporal frequency and 0.001° spatial resolution. SNSR uses the Simplified Simple Biosphere model to produce a prior estimate of SWE and then updates that prior using Landsat satellite snow cover estimates and a Bayesian geospatial statistical model. We chose to use SNSR because it is spatially continuous, observationally constrained, available at daily temporal resolution, and compared well to *in-situ* observations, with a mean uncertainty less than 3 cm compared with snow pillows and snow course.

The Parameter-elevation Relationships on Independent Slopes Model (PRISM) dataset^55,56^ is chosen as the reference T and P dataset. PRISM uses 10,000 + meteorological station observations to statistically interpolate to a 4-km resolution grid of the continental US (and a proprietary 800-m grid). Therefore, PRISM uses no physical models in its geospatial interpolation, instead it relies on geospatial statistical relationships between station derived climate variables and geospatial information such as atmospheric vertical layer location, coastal proximity, elevation, geographic location, orographic effectiveness, and topographic orientation. We use PRISM daily total P and daily mean T at 4 km resolution for our study. The daily mean T in PRISM is calculated from the average of the daily maximum and minimum T. To ensure consistency between model and reference, we also derive NA-CORDEX daily mean T using the average of maximum and minimum T.

Observational campaigns in the Sierra Nevada have shown that snowfall can occur between 0 and 3 °C^28,29^, with 90% of precipitation falling as snow at 0 °C, 50% at 1.5 °C, and 10% at 3 °C. Therefore, we derive reference S from PRISM daily P and T and assume that 100% of precipitation falls as snowfall below −0.375 °C with a linear increase in rainfall of 10% every + 0.375 °C up to 3.375 °C where 100% of precipitation falls as rainfall. We use the digital elevation model (DEM) data from Global 30 Arc-Second Elevation (GTOPO30)^57^ developed by the U.S. Geological Survey’s Center for Earth Resources Observation and Science as the reference topography, which is used to derive the elevation-modified T.

All reference data are first coarsened to 0.125° resolution, and then variables from model and reference are interpolated to a common 0.125°×0.125° grid. The model-reference comparison and the decomposition of the difference focus on 10 watershed regions in the California Sierra Nevada (Fig. 1) over 20 water years spanning OCT 1989 to SEP 2009.

### Uncertainties in quantitative decomposition

The uncertainties contained in the reference datasets and in our method lead to uncertainties in the resultant quantitative decomposition of SWE difference between model and reference datasets. We quantify reference data uncertainties using documented confidence intervals derived from the literature on each reference dataset. We propagate these uncertainties through the quantitative decomposition framework to provide the confidence intervals on each decomposed contribution found in Fig. 3. See the Supplementary Information for a detailed discussion of these uncertainties.

## Supplementary information

A quantitative method to decompose SWE differences between regional climate models and reanalysis datasets

## Data Availability

The datasets analyzed during the current study are publicly available at their source repositories or via the Department of Energy National Energy Research Scientific Computing Center at http://portal.nersc.gov/archive/home/a/arhoades/Shared/www/XU_2019.
