# Supplementary material for: A quantitative method to decompose SWE differences between regional climate models and reanalysis datasets
Source: Sci Rep. 2019 Nov 11;9:16520. doi: 10.1038/s41598-019-52880-5 (PMC6848092; doi:10.1038/s41598-019-52880-5)
Supplement: Supplementary file 1 — A quantitative method to decompose SWE differences between regional climate models and reanalysis datasets [file 41598_2019_52880_MOESM1_ESM.pdf]

Scientific Reports

Supplementary Information for

A quantitative method to decompose SWE differences between regional climate models and reanalysis datasets

Yun Xu[1,*], Andrew Jones[1], and Alan Rhoades[1]

[1]Lawrence Berkeley National Laboratory, Earth and Environment Sciences Area, Berkeley, CA, 94720, USA

[*]Corresponding author: Yun Xu (yunxu@lbl.gov)

Note: Uncertainties in the quantitative decomposition of model-reference difference in SWE

a. Uncertainties in SNSR SWE

The SNSR derived SWE was compared with 108 snow pillow and 202 snow course in-situ measurement sites throughout the Sierra Nevada (Margulis et al., 2016). For the 202 snow course sites, the mean error in SNSR was found to be less than 3 cm and the root-mean-square error was 13. In the 108 snow pillow measurement sites, the mean error in SNSR was 1 cm and the root-mean-square error was 11 cm. Averaged over all 310 measurements, the mean error is 2.3 cm, indicating the systematic error of SNSR is 2.3 cm; root-mean-square error is averaged 12.3 cm indicating a random error of 12.3 cm at each site. The random error at each site results in the standard error of the mean by $12.3/\sqrt{(310)} = 0.7$ cm, or 1.4 cm with a 95% confidence interval. Therefore, the total uncertainty is $\sqrt{(2.3^2 + 1.4^2)} \cong 3$ cm over the survey time and area when and where the mean SWE is 1.05 m, indicating a 3% uncertainty. This uncertainty affects the decomposition of model-reference difference of SWE where SNSR SWE is used in the calculation, i.e., row $\boldsymbol{\varepsilon}$ and $\boldsymbol{\varepsilon_M}$ in Fig. 3. In Table S1, we list each of the datasets used in Fig. 3 and include the true magnitudes of each variable along with their uncertainties. For example, reference SWE is 190 mm with 3% uncertainty, i.e., 190 (±6) mm.

b. Uncertainties in PRISM daily temperature and precipitation

The PRISM derived T were compared with in-situ measurements in the leeward side of the Sierra Nevada (Strachan and Daly, 2017). A random error with standard deviation of 1.67 °C was found. Our surveyed spatial-temporal extent covers about 3000 PRISM grids point over 3000 days. Therefore the random error only contributes to negligible error of the mean, which is $1.67/\sqrt{(3000 \times 3000)} < 0.001$ °C. However, a cold bias associated with topography was reported in PRISM daily T, with -0.75 °C bias in daily minimum T (Tmin) and -1.95 °C bias in daily maximum T (Tmax), or -1.35 °C in mean T. Further, we compared the PRISM and Livneh 2015 (L15) reanalysis dataset and found PRISM to be warmer than L15 by 1.1 °C in the windward side of Sierra Nevada. It is reasonable to believe the 95% confidence interval is below the upper limit of the systematic error (1.35 °C), which would result in a ± 69 mm uncertainty in snowfall. This uncertainty should be considered when comparing snowfall calculated from reference T and from modeled T. In Table S1, this is included in row REF in column $\widehat{\boldsymbol{S}}$, $\widehat{\boldsymbol{S}}_{\boldsymbol{T}}$, $\widehat{\boldsymbol{S}}_{\boldsymbol{T}''}$.

PRISM derived P was shown to have an estimated seasonal bias of 0-3% in North Carolina (Daly et al., 2017). In the Sierra Nevada, a -1% and -4% seasonal P bias was found for two reanalysis datasets that rescaled their estimates to the PRISM climatology (Lundquist et al., 2015). Given these two in-situ validation attempts of PRISM derived P, we assume a ±3% uncertainty due to systematic error in our surveyed area, which would lead to ±3% (± 10 mm) uncertainty in snowfall. This uncertainty should be considered when comparing snowfall calculated from reference P and from modeled P. Similar to PRISM daily T, the random error of PRISM daily P make negligible contribution to the standard error of the mean due to the large sample size. In Table S1, uncertainties in PRISM daily P is included in row REF in column $\widehat{\boldsymbol{S}}$, $\widehat{\boldsymbol{S}}_{\boldsymbol{P}}$, $\widehat{\boldsymbol{S}}_{\overline{\boldsymbol{P}}}$, $\widehat{\boldsymbol{S}}_{\boldsymbol{P}'}$.

c. Uncertainties about when precipitation occurs

We use daily total P and daily mean T to calculate the daily total snowfall in both reference and model datasets. The underlying assumption is that all precipitation occurs at daily mean T. However, precipitation could occur at any time of the day, and we do not know the exact distribution of P with T from our datasets. Therefore, we use a Monte Carlo method to estimate the associated uncertainty in snowfall, by assuming P occurs at a random T between Tmax and Tmin. The random T follows uniform distribution between Tmax and Tmin. It should be noted that the time of day when P occurs is not completely independent between grid cells, which means a reduced degree of freedom in random samples. To reduce the degree of freedom accordingly,

we assume the random T has the same relative distance between Tmin and Tmax on the same day at all grid points. Therefore, snowfall estimates are calculated using daily total P and the generated random T. We repeat the generation of random T and calculation of snowfall until the mean snowfall converges. The associated uncertainties in snowfall equals twice the standard deviation and represents a 95% confidence interval. This results in an 8 mm uncertainty in reference snowfall, and a 4-9 mm uncertainty (dependent on the amount of P and diurnal variability of T) in NA-CORDEX snowfall in column $\hat{\boldsymbol{S}}$, $\hat{\boldsymbol{S}}_{\boldsymbol{P}}$, $\hat{\boldsymbol{S}}_{\bar{\boldsymbol{P}}}$, $\hat{\boldsymbol{S}}_{\boldsymbol{P}'}$, $\hat{\boldsymbol{S}}_{\boldsymbol{T}}$, $\hat{\boldsymbol{S}}_{\tilde{\boldsymbol{T}}}$, $\hat{\boldsymbol{S}}_{\boldsymbol{T}''}$ and $\hat{\boldsymbol{S}}_{\boldsymbol{Th}}$ in Table S1.

d. Uncertainties about rain-snow partitioning

Snowfall in the Sierra Nevada has been shown to occur between 0 – 3 °C, with 90% precipitation falling as snow at 0 °C, and 10% at 3 °C (Lundquist et al., 2008; US Army Crops of Engineers, 1956). To assess the associated uncertainty with this observed estimate of rain-snow partitioning, we assume a random rain-to-snow percentage following uniform distribution between 0 and 1 at each grid cell and each day. We then apply a Monte Carlo method and found that the resultant uncertainty in the total snowfall is below 1 mm. This is assumed to be negligible.

e. Uncertainties in lapse rate

Lapse rates on the windward side of Sierra Nevada were found to range between 3.5 and 5.0 °C/km (Wolfe, 1992). Assuming the lapse rate is uniformly distributed between 3.5 and 5.0 °C/km, then 95% confidence interval for lapse rate is also between 3.5 to 5.0 °C/km. If we modify the lapse rate to 3.5 or to 5.0 °C /km, this changes $\hat{\boldsymbol{S}}_{\boldsymbol{T}''_{\boldsymbol{cdx}}}$ and $\hat{\boldsymbol{S}}_{\tilde{\boldsymbol{T}}_{\boldsymbol{cdx}}}$ by 1 to 11 mm in addition to the uncertainties caused by other factors. The total uncertainties are shown in Table S1.

f. Propagation of uncertainties

For variables influenced by more than one uncertainty, total uncertainty is the root sum square of each uncertainty. Therefore, the total uncertainty is a combined estimate of systematic errors and random errors with a 95% confidence interval.

We calculate the uncertainties in ablation (Table S1, column M) and in decomposed model-reference difference using the upper/lower bound in the 95% confidence interval of upstream features, so the uncertainties propagate to determine the uncertainty in the decomposition presented in Fig. 3.

Reference

Daly, C., et al. High-resolution precipitation mapping in a mountainous watershed: Ground truth for evaluating uncertainty in a national precipitation dataset. *Int. J. Climatol*. doi:10.1002/joc.4986 (2017).

Livneh B., et al. A spatially comprehensive, hydrometeorological data set for Mexico, the U.S., and southern Canada 1950-2013, Nature Scientific Data, 5:150042, doi:10.1038/sdata.2015.42 (2015).

Lundquist, J. D., et al. Rain versus snow in the Sierra Nevada, California: Comparing Doppler profiling radar and surface observations of melting level. *J. Hydrometeor.*, **9**(2), 194-211(2008).

Lundquist, J.D., et al. High-Elevation Precipitation Patterns: Using Snow Measurements to Assess Daily Gridded Datasets across the Sierra Nevada, California. *J. Hydrometeor.,* **16**, 1773–1792, https://doi.org/10.1175/JHM-D-15-0019.1 (2015)

Margulis, S. A., Cortés, G., Girotto, M., & Durand, M. A Landsat-Era Sierra Nevada Snow Reanalysis (1985–2015). *J. Hydrometeorol.,* **17**(4), 1203–1221, doi: 10.1175/JHM-D-15-0177.1 (2016).

Strachan, S, & C. Daly. Testing the daily PRISM air temperature model on semiarid mountain slopes. *J. Geophys. Res. Atmos*,**122**(11): 5697-5715 (2017).

US Army Corps of Engineers. Snow hydrology: Summary report of the snow investigations. (1956).

Wolfe, Jack A. An analysis of present-day terrestrial lapse rates in the western conterminous United States and their significance to paleoaltitudinal estimates. No. 1964. US Geological Survey, US, GPO; Books and Open-File Reports Section [distributor], (1992).

Table S1. Reference dataset (REF) and regional climate model simulated SWE, snowfall and ablation rate estimates. These variables are used for the decomposition of model-reference difference in SWE as defined in the Methods section. Total uncertainty at a 95% confidence interval is shown for each estimate in parentheses.

**Dataset**	$\widehat{SWE}$ (mm)	$\hat{S}$ (mm)	$\hat{S}_P$ (mm)	$\hat{S}_{\bar{P}}$ (mm)	$\hat{S}_{P\prime}$ (mm)	$\hat{S}_T$ (mm)	$\hat{S}_{\tilde{T}}$ (mm)	$\hat{S}_{T''}$ (mm)	$\hat{S}_{Th}$ (mm)	M (%)
**REF**	190 (6)	294 (70)	294 (13)	294 (13)	294 (13)	294 (69)	294 (13)	294 (69)	294 (8)	35 (22)%
**CRCM5 – 44**	59	121 (5)	209 (7)	227 (6)	271 (9)	265 (7)	240 (14)	310 (9)	215 (4)	51 (2) %
**CRCM5 – 22**	101	180 (6)	234 (8)	269 (7)	255 (9)	307 (7)	274 (9)	321 (6)	215 (4)	44 (2) %
**CRCM5 – 11**	117	196 (6)	244 (8)	292 (7)	246 (8)	307 (6)	287 (8)	313 (6)	215 (4)	40 (2) %
**CanRCM4 – 44**	25	98 (6)	156 (5)	170 (4)	269 (9)	292 (9)	240 (14)	339 (9)	215 (4)	74 (2) %
**CanRCM4 - 22**	67	153 (6)	176 (6)	205 (5)	253 (8)	350 (8)	274 (9)	363 (7)	215 (4)	56 (2) %
**RegCM4 - 44**	83	370 (6)	242 (8)	290 (7)	245 (8)	348 (9)	238 (14)	381 (7)	332 (4)	78 (1) %
**RegCM4 - 22**	253	544 (6)	325 (11)	393 (10)	243 (8)	388 (8)	277 (9)	390 (7)	332 (4)	53 (1) %
**WRF - 44**	44	172 (6)	245 (6)	247 (6)	291 (8)	297 (5)	268 (10)	321 (6)	215 (4)	75 (1) %
**WRF - 22**	85	242 (6)	283 (8)	288 (7)	289 (8)	333 (4)	285 (8)	339 (4)	215 (4)	65 (1) %

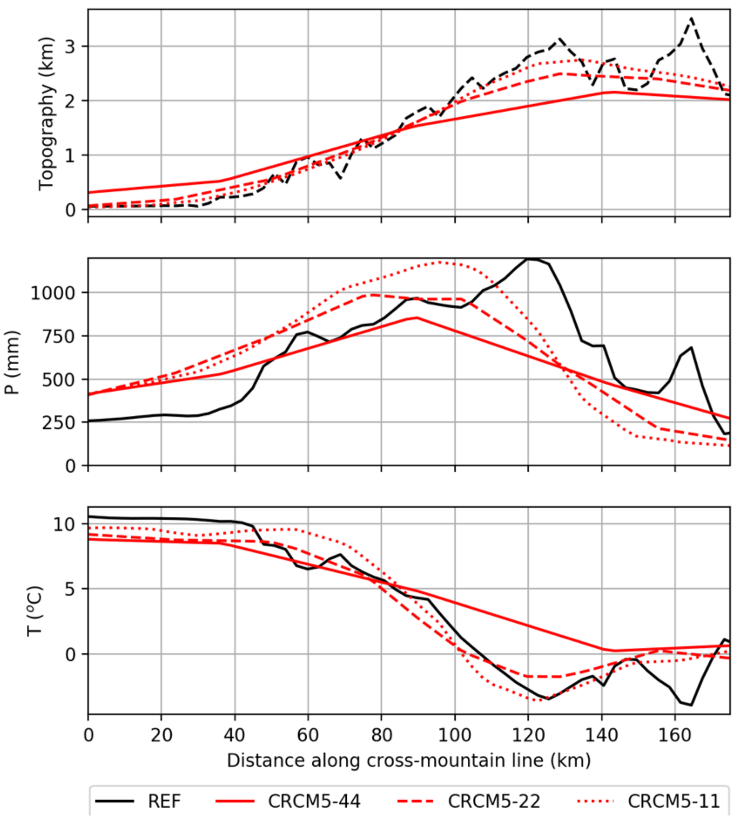

Figure S1. Land surface topography (in km), and 20-years mean winter precipitation (P, in mm) and temperature (T, in °C) from the reference dataset (REF) and CRCM5 model running at 0.44° (CRCM5-44), 0.22° (CRCM5-22) and 0.11° (CRCM5-11) spatial resolution. Data is linearly interpolated to the Sierra Nevada transect line as shown in Figure 1. P and T are temporally averaged over 20 winters between November and March from year 1985 to 2005.

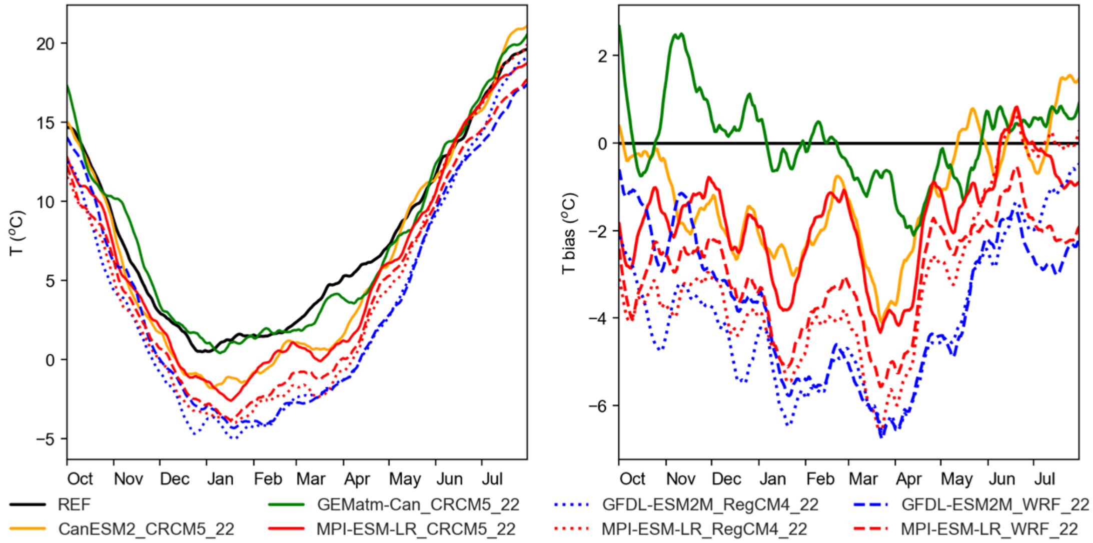

Figure S2. Air temperature from the NA-CORDEX regional climate model (RCM) simulations forced by global climate model (GCM), compared against the PRISM reference dataset. The plotted climatological mean spans 20-years from OCT 1985 to SEP 2005, averaged over the 10 California headwater regions. Simulations are named in the format of "GCM_RCM_resolution". Line-style indicates the RCM and color indicates the GCM used for boundary forcing.
